# Needle Arthroscopy Demonstrates High Sensitivity and Specificity for Diagnosing Intra-Articular Shoulder and Knee Pathology

**DOI:** 10.7759/cureus.33189

**Published:** 2022-12-31

**Authors:** Brittany DeClouette, Amy Birnbaum, Hilary Campbell, Andrew S Bi, Charles C Lin, Steven Struhl

**Affiliations:** 1 Department of Orthopedics, NYU (New York University) Langone Health, New York City, USA

**Keywords:** intra-articular shoulder pathology, rotator cuff tear, intra-articular knee pathology, meniscus tear, arthroscopy, needle arthroscopy

## Abstract

Introduction: Needle arthroscopy has been introduced in recent years as an alternative to magnetic resonance imaging (MRI) for the evaluation of knee and shoulder conditions. It has continued to evolve at a rapid rate with newer generation models making in-office integration simple. As MRI can sometimes prove inconclusive, it is worthwhile to consider these alternative options for evaluating musculoskeletal pathology as a primary diagnostic tool.

Purpose: The purpose of this study is to evaluate the specificity and sensitivity of needle arthroscopy in diagnosing intra-articular shoulder and knee pathology in a small case series of patients who ultimately underwent surgical arthroscopy.

Methods: A retrospective, single-centre, single-surgeon, cohort study was performed over a three-year period from August 2018 to June 2021. During this time, diagnostic needle arthroscopy was performed on patients with suspected shoulder or knee pathology based on MRI findings and clinical exams. These patients subsequently underwent standard surgical arthroscopy.

Results: Thirty-four patients were included in the study. There were 35 joints included, 25 shoulders and 10 knees, with a mean age of 41.88 +/- 11.32 years and BMI of 29.33 +/- 6.27 in the shoulder group and a mean age of 45.5 +/- 14.54 and BMI of 31.5 +/- 4.94 in the knee group. When evaluating shoulder pathologies, needle arthroscopy showed a sensitivity of 0.93 for rotator cuff tears, 1.00 for labral tears and 1.00 for loose bodies. Needle arthroscopy for the shoulder was found to be 100% specific for all shoulder pathologies. Needle arthroscopy for the knee was found to have a 1.00 sensitivity for detecting chondral defects and 0.80 sensitivity for meniscal tears. There were once again no false positive needle arthroscopy findings amongst the knee group.

Conclusion: Needle arthroscopy is an accurate diagnostic tool for the evaluation of intra-articular knee and shoulder musculoskeletal pathology. It can provide a potential solution for MRI-derived diagnostic inaccuracies that can lead to missed pathologies or unindicated procedures. It is less invasive than surgical arthroscopy and should be considered a useful tool in the armamentarium of orthopedic surgeons.

## Introduction

Needle arthroscopy has been introduced in recent years as an alternative to magnetic resonance imaging (MRI) for the evaluation of knee and shoulder conditions [1,2]. While MRI is still widely considered the gold standard for diagnosing these pathologies, recent studies have demonstrated that in-office needle arthroscopy can provide a more detailed and accurate diagnostic assessment of intra-articular pathologies than MRI [3,4]. Furthermore, multiple factors can affect the diagnostic accuracy of MRI, such as magnetic strength, study quality, and the interpreting physician’s level of experience. This alone makes having an additional diagnostic tool with potentially higher accuracy than MRI a powerful addition to the treating surgeon’s armamentarium.

Song et al. evaluated 185 knee MRIs, and concluded that 43% of the scans were inaccurate, with 18% of them being equivocal [5]. But what proves even more worrisome is that the literature has also shown that nearly 50% of orthopedic surgeons would operate on a patient with an inconclusive MRI [6]. Therefore, it is worthwhile to consider alternative options for evaluating musculoskeletal pathology as a primary diagnostic tool. Having an additional modality available to evaluate pathologies commonly seen in these two joints prior to pursuing a full surgical procedure can mitigate situations including, but not limited to, those where patients are unable to undergo MRI, or where MRI proves to be equivocal.

Needle arthroscopy is a relatively new technology that is evolving at a rapid rate. Multiple companies have now developed their own needle-based arthroscopy systems, which have already gone through multiple iterations. Overall, they are less invasive than surgical arthroscopy and can easily be performed in-office with an awake patient. The camera, light source, and connection cord are packaged into a single disposable unit, thus eliminating the need for sterilization between procedures. Additionally, these scopes omit the need for general anesthesia or fluid pump irrigation systems while still allowing for direct visualization and immediate diagnostic information [3,6,7]. Furthermore, newer second-generation needle arthroscopy devices report 3-5x higher image resolutions than older devices [8].

The purpose of this study is to evaluate the sensitivity and specificity of second-generation needle arthroscopy in a small cohort of patients with suspected shoulder or knee intra-articular pathology who ultimately underwent surgical arthroscopy. We hypothesized that needle arthroscopy would have high sensitivity and specificity in diagnosing knee and shoulder pathology.

## Materials and methods

Patient selection

A retrospective, single-center, single-surgeon, cohort study was performed over a three-year period from August 2018 to June 2021. During this time, diagnostic needle arthroscopy was performed in patients with suspected shoulder or knee pathology based on clinical examination and MRI findings. Subsequently, a chart review was conducted on all patients who then went on to surgical arthroscopy. Indications for MRI varied for each patient based on their symptoms, the mechanism of injury, and which joint was affected. However, in general for atraumatic injuries, MRI was performed after six weeks of non-operative treatment failed to provide improvement. Indications for needle arthroscopy were inconclusive MRI images and/or radiology reads. Indications for arthroscopy were continued pain or symptoms in the knee or shoulder and confirmation of intra-articular pathology on diagnostic needle arthroscopy. Any patient who did not receive diagnostic needle arthroscopy followed by surgical arthroscopy was excluded from the study. 

Data collection

Patient demographic information, including age, gender, joint location, and body mass index (BMI), as well as operative reports and photographs for needle arthroscopies and surgical arthroscopies, were collected for all patients. All pathologic findings from needle and surgical arthroscopy operative reports and intra-operative photos were recorded by two separate authors, that were not the primary surgeon, and categorized into relevant findings. Several pathologies were excluded from this study, including but not limited to, extra-articular pathologies such as lateral collateral ligament (LCL) or medial collateral ligament (MCL) injuries, and pathologies that when isolated are not surgical, such as subacromial bursitis.

Surgical technique

All needle arthroscopies were performed in the operating room setting at either a large academic urban medical center or a privately owned surgery center. Each procedure utilized one of two second-generation, high-resolution needlescope devices, either the IntraVu MIDASVu arthroscope (32 cases) (IntraVu, Inc., Redwood City, California, United States) or the Arthrex® NanoScope™ (two cases) (Arthrex, Inc., Naples, Florida, United States), and gas insufflation instead of saline, as previously described [9].

**Figure 1:**
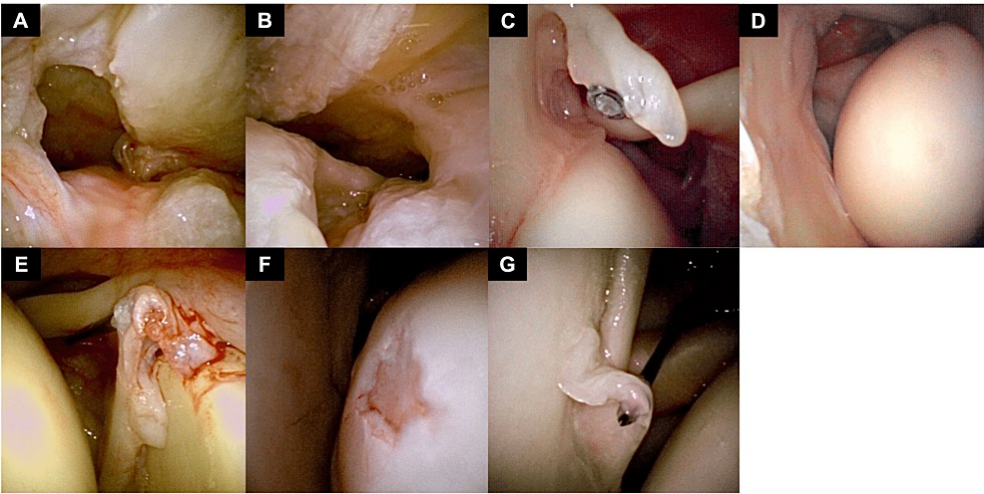
Needle Arthroscopy Visualization of Shoulder Pathology (A) Articular-sided view of a complete rotator cuff tear; (B) Bursal-sided view of complete rotator cuff tear; (C) Partial rotator cuff tear; (D) Unstable shoulder; (E) Type 2 SLAP tear; (F) Humeral head chondral lesion; (G) Anterior labral tear SLAP: Superior Labrum, Anterior to Posterior

All shoulder needle arthroscopies were positioned using the beach chair position, using local anesthesia. The needle arthroscope was inserted in the standard posterior portal and 20 cc of air was injected into the glenohumeral joint for visualization (Figures 1A, 1C, 1D, 1E, 1F, 1G).

An 18-gauge spinal needle placed into the anterior portal was used to visualize or probe relevant intra-articular pathology. After a systematic diagnostic arthroscopy was performed, the needle arthroscope was removed and then placed into the subacromial space, and another 20 cc of air was injected (Figure 1b), using an 18-gauge spinal needle from the lateral portal as a probe. At the end of the procedure, air was removed from both the subacromial space and glenohumeral joint, and Marcaine was injected for post-procedural pain control. 

**Figure 2:**
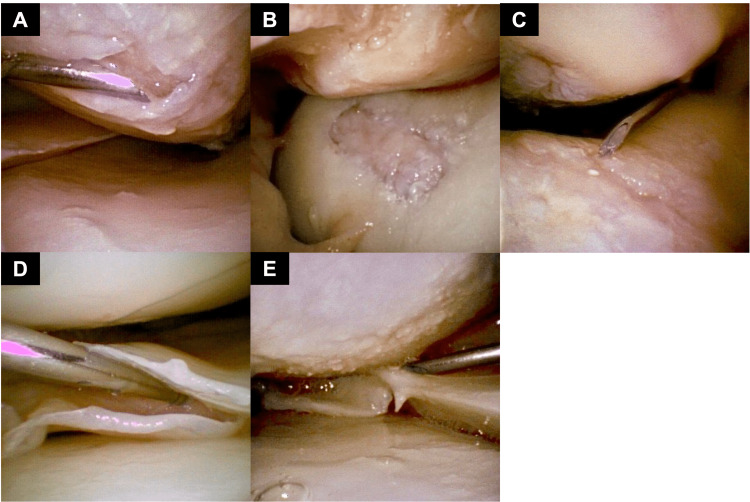
Needle Arthroscopy Visualization of Knee Pathology (A) Medial femoral condyle chondral lesion; (B) isolated trochlea lesion; (C) patella and trochlea lesions; (D) lateral meniscus horizontal tear; (E) medial meniscus tear

All knee needle arthroscopies were positioned supine with the operative extremity in a leg holder and a break in the bed. Intravenous sedation was provided by anesthesia, and a tourniquet was inflated to 250 mmHg. Local anesthesia was infiltrated into the standard anterolateral portal soft tissue. In cases where an effusion was present, 60 cc of saline was injected into the knee and then immediately removed. Finally, the needle arthroscope was inserted into the anterolateral portal followed by 50 cc of air injected into the suprapatellar space via the side port of the scope. A standard diagnostic arthroscopy was performed, examining the suprapatellar pouch, patellofemoral joint, medial and lateral gutters, medial and lateral compartments, and intercondylar notch (Figure 2A, 2B, 2C, 2D, 2E).

Relevant intra-articular pathology was probed with an 18 gauge spinal needle from the anteromedial portal. Following the procedure, the air was aspirated out of the joint, and 10 cc of Marcaine was injected for post-procedural pain control.

All surgical arthroscopies were performed at either a large academic urban medical center or a privately owned surgery center. All shoulder arthroscopies were performed in the beach chair position under interscalene regional nerve block or general anesthesia. All knee arthroscopies were performed in the supine position under regional anesthesia with sedation. Tourniquet exsanguination to 250 mmHg was used for all knees, with a break in the bed and leg holder for positioning.

Statistical analysis

Sensitivities and specificities were calculated for each pathology comparing needle arthroscopy to surgical arthroscopy as the gold standard. All statistical analysis was performed in R (version 3.6.2; R Foundation for Statistical Computing, Vienna, Austria). Positive predictive value (PPV) and negative predictive value (NPV) were compared using a weighted generalized score statistic and Cohen’s Kappa statistics were calculated.

## Results

Overall results

Thirty-four total patients who underwent needle arthroscopy and then formal surgical arthroscopy were included in the study. There were 35 joints included, 25 shoulders and 10 knees, with a mean age of 41.88 +/- 11.32 years and BMI of 29.33 +/- 6.27 in the shoulder group and a mean age of 45.5 +/- 14.54 and BMI of 31.5 +/- 4.94 in the knee group (Table 1).

**Table 1:** Demographics of Included Patients

	Shoulders	Knees
N	25	10
Age (mean (SD))	41.88 (11.32)	45.5 (14.54)
Sex = M (%)	15 (60.0)	7 (70.0)
BMI (mean (SD))	29.33 (6.27)	31.5 (4.94)
Laterality (Right)	11 (44.0)	6 (60.0)

There were no patient-related or device-related complications that occurred during this study period.

Shoulder-specific pathology

When evaluating shoulder pathologies, needle arthroscopy showed a sensitivity of 0.93 for rotator cuff tears, 1.00 for labral tears, and 1.00 for loose bodies. Needle arthroscopy for the shoulder was found to be 100% specific for all shoulder pathologies (Table 2).

**Table 2:** Shoulder Pathology Sensitivities and Specificities

Pathology	Needle Arthroscopy Sensitivity	Needle Arthroscopy Specificity
Rotator Cuff Tears	0.93 (0.81-1.00)	1.00 (1.00-1.00)
Labral Tears	1.00 (1.00-1.00)	1.00 (1.00-1.00)
Loose Body	1.00 (1.00-1.00)	1.00 (1.00-1.00)

There were no false positive needle arthroscopy findings in any of the studies. The PPV of needle arthroscopy was 1.00 (1.00-1.00) in the shoulder group and the NPV was 0.98 (0.95-1.00) (Table 3).

**Table 3:** PPV and NPV of Needle Arthroscopy PPV: Positive predictive value; NPV: Negative predictive value

	PPV	NPV
Shoulder	1.00 (1.00-1.00)	0.98 (0.95-1.00)
Knee	1.00 (1.00-1.00)	0.93 (0.84-1.00)

When compared to surgical arthroscopy, needle arthroscopy for the knee was found to have a 1.00 sensitivity for detecting chondral defects and 0.80 sensitivity for meniscal tears. There were once again no false positive needle arthroscopy findings amongst the knee group. Specificity of needle arthroscopy was 1.0 for both meniscus and chondral pathology (Table 4).

**Table 4:** Knee Pathology Sensitivities and Specificities

Pathology	Needle Arthroscopy Sensitivity	Needle Arthroscopy Specificity
Meniscal Tears	0.80 (0.45-1.00)	1.00 (1.00-1.00)
Chondral Defects	1.00 (1.00-1.00)	1.00 (1.00-1.00)

The PPV was 1.00 (1.00-1.00) for needle arthroscopy in the knee group. NPV value was 0.93 (0.84-1.00) (Table 3). When evaluated via Cohen’s Kappa statistic, needle arthroscopy had high inter-rater reliability in both the shoulder and knee group at 0.922 and 0.733, respectively (Table 5).

**Table 5:** Inter-rater Reliabilities Between Needle Arthroscopy and Surgical Arthroscopy

	Inter-rater reliability
Shoulder	k = 0.922
Knee	k = 0.733

## Discussion

This study evaluated a small case series of patients who underwent needle arthroscopy with a second-generation needlescope device, and eventually underwent surgical arthroscopy. The most significant finding was the high sensitivity and specificity demonstrated in needle arthroscopy. The specificity was 100% across all joints and pathologies identified. This exhibits the excellent utility of needle arthroscopy to effectively rule in intra-articular pathologies in the knee and shoulder. In combination with high sensitivity, these results argue that needle arthroscopy is an accurate diagnostic tool that can be used alone or in conjunction with advanced imaging.

Prior studies have evaluated the sensitivity and specificity of MRI as a diagnostic modality for intra-articular knee and shoulder pathology, and it is well established that this is the gold standard for making these diagnoses. In a systematic review by Phelan et al., MRI was found to have sensitivities and specificities in the high 80s to 90s for both meniscus and anterior cruciate ligament (ACL) tears [10], which is equal to or lower than those of our study. Additionally, there have been a few studies directly comparing the two modalities. Wagner et al., examined the shoulders of 50 patients who underwent MRI, needle arthroscopy, and surgical arthroscopy [7]. They found similar accuracies between MRI and needle arthroscopy, with needle arthroscopy having slightly higher specificity but lower sensitivity than MRI. They used the Mi-Eye™ (Trice Medical, Malvern, Pennsylvania, United States), and performed their diagnostic needle arthroscopy at the same anesthetic event in the operating room. Another similar study [3], compared the accuracy, sensitivity, and specificity of MRI and needle arthroscopy to surgical arthroscopy focusing specifically on knee pathology. They found that needle-based arthroscopy performed in the office was statistically equivalent to surgical arthroscopy in the operating room for diagnosing intra-articular knee joint pathologies. These studies further corroborate our findings and even suggest that needle arthroscopy could be used alone instead of MRI for making diagnoses in suspected intra-articular knee or shoulder conditions.

It is important to note that the accuracy of the needlescope in this study tended to be higher than that of previously published data, with no false positives identified in either knee or shoulder pathology. The hypothesized reason for this improvement in accuracy is two-fold. First, all needle arthroscopy cases in this study used second-generation needlescope devices, which have improved technological specifications when compared to older first-generation models. The second-generation devices also note 3-5x higher image resolutions than older devices. Secondly, the needle arthroscopy protocol used in this study involved using air instead of saline as the viewing medium. The most commonly used standardized diagnostic approach to the knee uses methodology described by McMillan et al. [6], which involves 30cc of saline to visualize the joint space. It can be argued that using air instead of saline likely contributed to the high diagnostic accuracy shown in our study, as the refractive index of air compared to water provides a wider field of view, which is particularly advantageous when using a zero-degree arthroscope. Additionally, clarity is improved as there is no floating debris to contend with. Currently, all needlescopes are zero-degree scopes.

In-office needle arthroscopy presents a promising opportunity for improved patient care, by accurately diagnosing various intra-articular pathologies in the shoulder and the knee. Additionally, cost analyses reported in the literature have demonstrated a net cost savings of over 100 million dollars to the United States system when using needle arthroscopy over MRI [11], as unnecessary surgical arthroscopy cases can be avoided. It is important to note that while overall a very safe procedure, there are some potential adverse outcomes. For example, there are reports of vasovagal events from in-office needle arthroscopy as well as hemarthroses that cannot be successfully flushed with running saline. Such events outside the monitored setting of an operating room can present challenges to the physician and office staff in creating a safe environment for the patient. Furthermore, the time it takes to successfully perform needle arthroscopy varies from patient to patient, which can make it difficult to incorporate into the office setting [6,12]. For these reasons, it is preferential to use the needlescope under monitored settings, with a combination of local anesthesia and in certain cases, mild intravenous sedation, in order to perform the safest and most accurate procedure possible. Although unavailable to the senior author for this study, it is believed that a limited-use “procedure room” would be the most cost-effective for more widespread use of this technique.

Limitations

This study is not without limitations. This was a retrospective, single-surgeon study. Given the nature of a single surgeon study, there was likely a learning curve over the three-year period, which may have influenced results, and could have also added bias to the interpretation. As previously mentioned, the needle arthroscopy technique in this study was performed in the operating room and had the benefit of intravenous sedation, and positioning devices such as a beach chair arm holder, or leg holder, and thus these results may not be as applicable to in-office diagnostic needle arthroscopy. This study also did not include an MRI comparison and focused solely on the specificity and sensitivity of the needle arthroscopy alone, which eliminates the ability to make any conclusions about inferiority. Finally, the study was limited to the shoulder and knee, and more specifically only intra-articular pathology. Thus, translation to other joints that commonly undergo needle arthroscopies, such as wrists and ankles, has yet to be determined.

## Conclusions

Needle arthroscopy is an accurate diagnostic tool for the evaluation of intra-articular knee and shoulder musculoskeletal pathology. It can provide a potential solution for MRI-derived diagnostic inaccuracies that can lead to missed pathologies or unindicated procedures. It is less invasive than surgical arthroscopy and should be considered a useful tool in the armamentarium of orthopedic surgeons. 
